# Supplementary material for: Examining the role of systemic inflammation as a mediator of the glycaemia-brain volume associations in women
Source: PLoS One. 2026 Mar 10;21(3):e0329046. doi: 10.1371/journal.pone.0329046 (PMC12974826; doi:10.1371/journal.pone.0329046)
Supplement: S1 File — Contains supplementary figures and tables detailing mediation models and inflammation-based pathway analyses relating glycaemic markers to brain volume outcomes. (DOCX) [file pone.0329046.s001.docx]

**Supplementary material:**

Latent variable analysis

For the latent model of inflammatory markers, there was evidence of a total and direct effect of with HbA_1c_ and glucose on WBV, but no total or direct effect on GM or WM volumes (Supplementary Table 2). There was no evidence of indirect effects observed between HbA_1c_ or glucose on WBV, GM, or WM volumes through the inflammatory pathway.

Model checks using several fit indices were used to determine the adequacy of the latent variable model. The Chi-square test was significant (p = 0.003), indicating that the model was misspecified to some extent. This result is not unexpected, given the sensitivity of the Chi-square test to sample size. The RMSEA value was 0.08, which is at the upper limit of what is considered a “reasonable fit”, suggesting that there is some error in approximation, but it is not excessively poor. The CFI value was 0.9, which falls within the range of acceptable fit (0.90 and 0.95 are deemed acceptable). In summary, these fit indices present a mixed picture of the model's adequacy: the significant Chi-square test and the RMSEA value suggest some limitations in model fit, while the CFI indicates an acceptable fit.
