# Supplementary figures and images for: Examining the role of systemic inflammation as a mediator of the glycaemia-brain volume associations in women
Source: PLoS One. 2026 Mar 10;21(3):e0329046. doi: 10.1371/journal.pone.0329046 (PMC12974826; doi:10.1371/journal.pone.0329046)

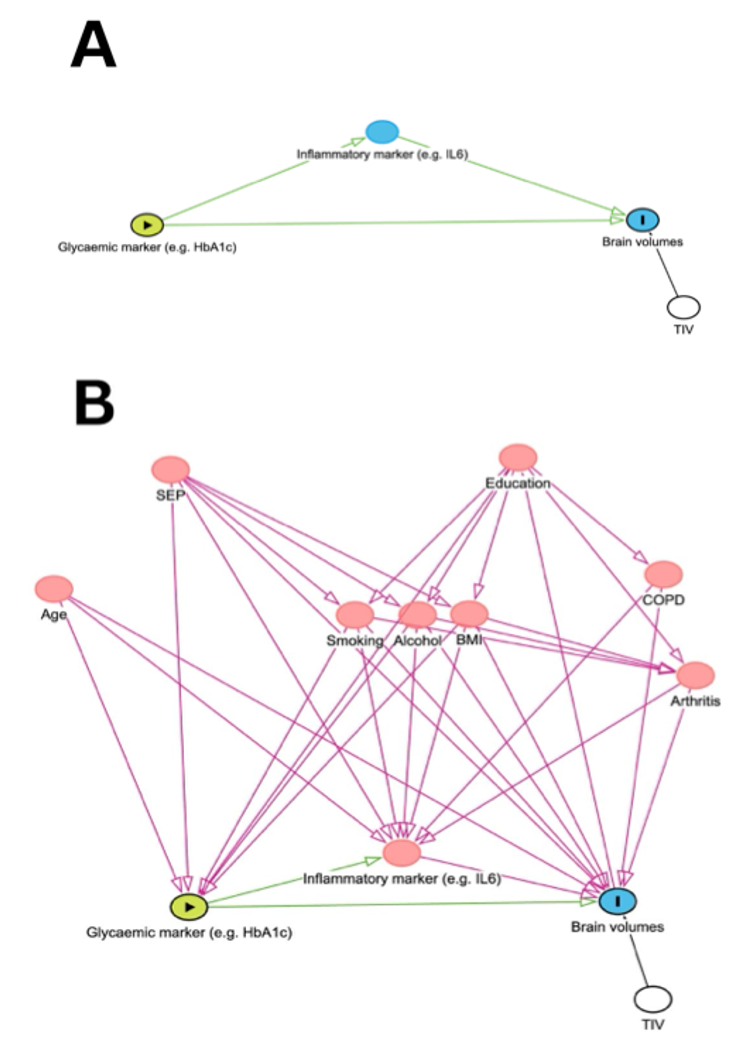

Supplement: S1 Fig — B) Fully confounder-adjusted model. Adjustments were made for both exposure-mediator and mediator-outcome relationships. (PNG) [file pone.0329046.s002.png]

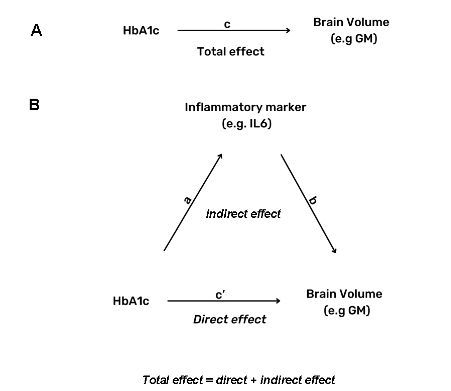

Supplement: S2 Fig — A) shows the assumed (total) effect, c. B) the total effect decomposed into a direct, c’ and indirect (mediated via a and b) effect. (PNG) [file pone.0329046.s003.png]
