# Supplementary material for: Examining the role of systemic inflammation as a mediator of the glycaemia-brain volume associations in women
Source: PLoS One. 2026 Mar 10;21(3):e0329046. doi: 10.1371/journal.pone.0329046 (PMC12974826; doi:10.1371/journal.pone.0329046)
Supplement: S1 Table — The total effects, direct, indirect effects, exposure-mediator are presented. β, Cl and p-values are presented. (DOCX) [file pone.0329046.s004.docx]

|  |  |  | Whole brain volumes (WBV) | | | | Grey matter volumes (GM) | | | | White matter volumes (WM) | | | | |
| --- | --- | --- | --- | --- | --- | --- | --- | --- | --- | --- | --- | --- | --- | --- | --- |
|  |  | Path | **β** | **95% CI** | | **p** | **β** | **95% CI** | | **p** | **β** | **95% CI** | | **p** |  |
| HbA_1c_ | Total effect | c | -1.1 | -2.1 | -1.0 | 0.03 | -0.2 | -0.7 | 0.3 | 0.3 | -0.2 | -0.6 | 0.2 | 0.3 |  |
|  | Direct effect | c' | -0.5 | -1.7 | 0.6 | 0.3 | -0.2 | -0.7 | 0.3 | 0.3 | 0.01 | -0.5 | 0.5 | 0.9 |  |
|  | Indirect effect |  | -0.5 | -1.3 | 0.1 | 0.1 | -0.02 | -0.1 | 0.1 | 0.7 | -0.2 | -0.5 | 0.08 | 0.1 |  |
|  | Exposure-mediator | a | -0.5 | -1.3 | 0.1 | 0.1 | -0.02 | -0.1 | 0.1 | 0.7 | -0.2 | -0.5 | 0.08 | 0.1 |  |
|  | Mediator-outcome | b | 1 Constrained | | | | 1 Constrained | | | | 1 Constrained | | | | |
|  |  | Path | **β** | **95% CI** | | **p** | **β** | **95% CI** | | **p** | **β** | **95% CI** | | **p** |  |
| Glucose | Total effect | c | -6.5 | -11.4 | -1.6 | 0.009 | -0.1 | -0.3 | 1.2 | 0.3 | -2.3 | -4.2 | -0.3 | 0.03 |  |
|  | Direct effect | c' | -5.3 | -10.3 | -0.02 | 0.04 | -0.1 | -3.2 | 1.4 | 0.4 | -1.9 | -3.9 | 0.2 | 0.08 |  |
|  | Indirect effect |  | -1.2 | -3.1 | 0.7 | 0.2 | -0.07 | -1.4 | 1.3 | 0.9 | -0.4 | -1.2 | 0.3 | 0.2 |  |
|  | Exposure-mediator | a | -1.2 | -3.1 | 0.7 | 0.2 | -0.07 | -1.4 | 1.3 | 0.9 | -0.4 | -1.2 | 0.3 | 0.2 |  |
|  | Mediator-outcome | b | 1 Constrained | | | | 1 Constrained | | | | 1 Constrained | | | | |
