# Supplementary material for: Examining the role of systemic inflammation as a mediator of the glycaemia-brain volume associations in women
Source: PLoS One. 2026 Mar 10;21(3):e0329046. doi: 10.1371/journal.pone.0329046 (PMC12974826; doi:10.1371/journal.pone.0329046)
Supplement: S2 Table — Shown are the total effect (c), direct effect (c′), indirect effect (ab), and component paths a (exposure→mediator) and b (mediator→WBV), with β coefficients, 95% confidence intervals (CI), and p-values (p). For both NLR and SII, total and direct effects were negative and statistically significant, whereas indirect effects were not significant, providing little evidence that NLR or SII mediates the exposure–WBV association. (DOCX) [file pone.0329046.s005.docx]

| **Whole brain volumes (WBV)** | | | | | | | | | |
| --- | --- | --- | --- | --- | --- | --- | --- | --- | --- |
|  |  | NLR | | | | SII | | | |
|  | Path | β | 95% CI | | p | β | 95% CI | | p |
| Total effect | c | -6.64 | -15.07 | -1.78 | 0.012 | -6.74 | -15.18 | -1.69 | 0.023 |
| Direct effect | c' | -6.42 | -14.99 | -1.78 | 0.011 | -6.63 | -15.25 | -1.99 | 0.022 |
| Indirect effect | ab | -0.24 | -2.45 | 1.97 | 0.831 | -0.11 | -2.34 | 2.12 | 0.901 |
| Exposure-mediator | a | 0.061 | -0.14 | 0.27 | 0.554 | 43.46 | -13.5 | 100.4 | 0.135 |
| Mediator-outcome | b | -3.963 | -5.54 | 1.78 | 0.214 | -0.003 | -0.01 | 0.01 | 0.93 |
